# Fermented cottonseed meal improves production performance and reduces fat deposition in broiler chickens

**DOI:** 10.5713/ajas.20.0571

**Published:** 2020-11-25

**Authors:** Jun Li Niu, Lian Qing Wei, Yuan Qing Luo, Wen Ting Yang, Qi Cheng Lu, Xin Xia Zheng, Yu Jie Niu, Wen Sheng, Hong Cheng, Wen Ju Zhang, Cun Xi Nie

**Affiliations:** 1 College of Animal Science and Technology, Shihezi University, Xinjiang 832000, China

**Keywords:** Fermented Feedstuffs, Growth Performance, Carcass Trait, Abdominal Fat, Adipocyte Surface, Poultry

## Abstract

**Objective:**

This study was conducted to investigate the effect of fermented cottonseed meal (FCSM) on growth performance, carcass traits, and fat deposition in white-feather broiler chickens.

**Methods:**

A total of 480 male one-day-old white-feather broiler chickens were selected randomly and divided into four groups with six replicates of 20 chickens in each. The experimental chickens were fed diets including 3%, 6%, or 9% FCSM fermented by *Candida tropicalis* until 42 days old. In the experiment, the chickens of the control group were fed soybean meal.

**Results:**

FCSM supplementation linearly decreased the feed conversion ratio from d 15 to 21 and d 36 to 42, respectively (p<0.05). The percentage of carcass and semi-eviscerate increased in response to dietary FCSM supplementation at d 21 (p<0.05). The percentage of eviscerated and semi-eviscerate of 3FCSM was higher than that in other groups at d 35 (p<0.05). At the age of 42 d, the percentage of carcass increased in a quadratic way among increasing FCSM in diets (p<0.05). The subcutaneous fat thickness linearly decreased with the increasing levels of FCSM at d 21 (p<0.05). Gompertz and Logistic functions provided a better fit on abdominal fat and subcutaneous fat, respectively. The best fitted equation predicted that the maximum growth rate of abdominal fat weight and subcutaneous fat thickness occurred at d 28. FCSM had no significant effects on the shape of growth curve of abdominal fat weight and subcutaneous fat thickness, but reduced the height of the curve. Birds receiving the 6FCSM diet for 21 d had smaller adipocyte surface and lower serum glucose as well as triglyceride concentration.

**Conclusion:**

FCSM is beneficial for broiler chickens as it positively affects their growth and carcass in addition to altering their fat deposition.

## INTRODUCTION

Cottonseed meal (CSM) is generally recognized as an alternative protein feedstuff for animals. However, the application of CSM as a feed ingredient in animal rations is limited due to its gossypol (a toxic polyphenolic pigment) content, crude fiber and low lysine level [1]. But the fermented cottonseed meal (FCSM) is a mixture of solid CSM with liquid phases and inoculated with beneficial microorganisms (e.g., *Lactobacillus* spp. and *Candida tropicalis* [*C. tropicalis*]). Microbial fermentation is currently considered as one of the most effective ways to reduce anti-nutritional factor in CSM such as free gossypol [2,3]. In addition, the metabolic activities of microorganisms during fermentation may produce enzymes, vitamins, and some unknown compounds into the CSM [1]. All these have a significant effect on the growth and health of animals [4]. Improved nutritional characteristics are seen in CSM fermented by microbes [5]. It happens because of variety of essential nutrients such as vitamins, small-size peptides, oligosaccharides and amino acids are formed during fermentation [6], extending the use of CSM in animal husbandry.

In the recent time there is a high demand for chicken production as a source of meat. So, to the commercial broiler chicken breeding farms the primary goals are to achieve faster growth, higher feed efficiency and more breast muscle yield in chickens. But the lack of appropriate feed resources is a bottleneck. On the other hand, in broiler chicken farms a rapid increase in the growth rate of broiler chickens has been accompanied by an increase in the deposition of body fat [7]. Fat has been considered as a by-product of very little commercial value [8]. Moreover, excessive fat deposition can increase feed cost and reduce feed conversion efficiency [9]. From the aforementioned drawbacks, it is clear that reducing excessive fat deposition is the most primary task for broiler chicken farms and consumers. In our previous studies, we found that FCSM regulates lipid metabolism by affecting the expression of the lipid-related gene and altering multiple endogenous metabolites [7]. Other studies have also proven that fermented products have the potential to decrease fat deposition and improve lipid profiles [10,11]. It is well known that the amount and rate of fat deposition in different parts of broiler chickens are variable. The abdominal fat is the highest and fastest than other tissues. While the subcutaneous fat is lower and deposits earlier than the abdominal fat. Different sex, breed and strain of broiler chicken have different regulatory mechanism of fat deposition. Despite the confirmed benefits of dietary FCSM, there is still little information available on the effect of FCSM on the fat deposition rate of broiler chickens.

In our previous study, *C. tropicalis* was found very effective in detoxifying free gossypol (FG) and in the improvement of the nutritional value of CSM [12]. Regarding fat deposition white-feather broiler chicken is a typical breed. However, few studies conducted on the effects of FCSM fermented by *C. tropicalis* on fat deposition in white-feather broiler chickens. Therefore, the objective of this study was to investigate the effect of FCSM fermented by *C. tropicalis* on the production performance, carcass traits as well as the growth curve of abdominal fat and subcutaneous fat in white-feather broiler chickens.

## MATERIALS AND METHODS

All animal works in this paper was conducted according to the relevant national and international guidelines. Animal care for the experiment complied with the regulations for the Animal Welfare Committee of Shihezi University (Xinjiang, China) (Ethical code: A2017-060-01).

### Substrate preparation and fermentation

Fermenting organism *C. tropicalis* strain was provided by the Feed Science Institute of Zhejiang University (Hangzhou, China). Fermentation substrates namely, CSM, wheat bran and corn flour were obtained from the Shihezi district (Xinjiang, China). The fermentation was carried out according to the process modified method [2]. The mixed ratio of CSM, corn flour, and wheat bran was 90:5:5 and the ratio of substrate and water was 1:0.8. The mixed substrate was then autoclaved at 121°C for 20 min. The substrate was taken out, cooled and each kilogram of substrate was mixed thoroughly with 80 mL of *C. tropicalis* (10^8^ cells/mL). The mixture was fermented in an incubator at 30°C for 48 h. After fermentation, the substrate residue was dried at 40°C for 48 h in a drying cabinet. The dried substrate was ground to 0.2 mm grain size and refrigerated until those were added to the diets.

### Animals and experimental design

A total of 480 male Cobb broiler chickens (500) were purchased at d 1 from Tengfei Poultry Industry Co., Ltd. (Henan, China). The broiler chickens were randomly assigned into four diets containing different levels of FCSM (0%, 3%, 6%, and 9%), named as 0FCSM, 3FCSM, 6FCSM, and 9FCSM. Each treatment included 6 replicates of 20 broiler chickens. Broiler chickens were raised in wire-floored cages (320×320× 90 cm). Each cage has three levels with five cages in each levels and four birds in each cages. The experiment was continued for 42 days. The broiler chickens were provided feed and water *ad libitum* throughout the experiment. The broiler chickens house was kept illuminated for 24 h with constant-light regimen during the whole trial period. Temperature at d 1 was 33°C with a decreasing rate of 5°C per week until the temperature reached to 23°C. Ventilation within the room was maintained by exhaust fans. Excreta was removed every day manually. The diets were prepared according to the Nutrient Requirements of Poultry (NRC, 1994). The experimental diets were formulated in mash form and the composition and nutrient content of which have been present in Table 1. While the amino acid profiles of FCSM and soybean meal were published in our previous study [13]. The nutritional composition of CSM, FCSM, and soybean meal are presented in Table 2.

### Collecting sample and assaying related index

#### Chemical composition

Unfermented CSM and FCSM by *C. tropicalis* from the experiment were analyzed for dry matter (DM), ether extract, crude protein (CP) and crude ash (Ash) content following the standard method of AOAC International (AOAC, 2000). Neutral detergent fiber (aNDFom) and acid detergent fiber (ADFom) were determined using heat stable amylase and expressed inclusive of residual ash [14]. The contents of FG were tested according to the standard method of the AOCS (AOCS, 2009).

#### Growth performance

In present study, the body weight was monitored weekly and the feed intake was recorded daily on a replicate basis. This data was used to calculate the average daily feed intake (ADFI), average daily gain (ADG) and feed conversion ratio (FCR, ADFI:ADG). The feed conversion was calculated and corrected for mortality. Mortality during the subsequent periods was based on the number alive in the remaining pens at the start of each period. The diet cost per kg = ∑(the cost of each diet ingredient per kg). The cost of diet per kg of broiler meat produced = total cost of feed intake/total weight gain.

#### Carcass trait

From the experimental broiler chickens, one individual broiler chicken from each replicate group was slaughtered by cervical dislocation for carcass measurements per week in the study site. By collecting the weight and carcass weight, the weight of total eviscerated and semi-eviscerated yield, abdominal fat, liver, the breast and thigh muscles were also taken separately. The broiler chickens were bled for 3 min and had feathers removed before obtaining the carcass weight. Eviscerate yield, which included the lungs and kidneys, but excluded all internal organs, head, and feet, were then weighed. The trachea, esophagus, craw, intestine, spleen, pancreas, gallbladder, and reproductive organs were removed before taking semi-eviscerated yield. The carcass traits were calculated as follows: carcass (%) = 100×(carcass weight/live weight); eviscerated (%) = 100×(total eviscerated yield weight/carcass weight); semi-eviscerated (%) = 100×(semi-eviscerated yield weight/carcass weight); and tissue (%) = 100×(tissue weight/total eviscerated yield weight).

#### Fat deposition curve

Six broiler chickens were randomly selected from each treatment (one broiler chicken from each replicate) and were slaughtered weekly. The abdominal fat pads (adipose tissue surrounding the gizzard, bursa of Fabricus, cloaca, and adjacent muscles) were collected and weighed. Meanwhile, the subcutaneous fat thickness was determined using a Vernier caliper. The relative growth coefficients of abdominal fat weight and subcutaneous fat thickness were calculated as follows:

$$\text{Y}=2\times ({\text{W}}_{2}-{\text{W}}_{1})/({\text{W}}_{2}+{\text{W}}_{1})$$

Where: Y is the relative growth coefficients, W_1_ is the initial weight or thickness of the week, W_2_ is the final weight or thickness of the week.

The Logistic [15], Gompertz [16], and Bertalanffy [17] growth models were used to fit the data of abdominal fat weight and subcutaneous fat thickness. The mathematical equation of these growth models has been shown in Table 3.

#### Histopathological analysis

On 21 d, four birds from each of the four treatments were randomly elected and abdominal fat collected. Abdominal fat was fixed in 4% paraformaldehyde. After washing in running water and dehydration in gradient ethanol, samples were saturated and embedded with paraffin. The 5-μm-thick sections were stained with hematoxylin-eosin and viewed under a light microscope (Leica DM LB2, Wetzlar, Germany) equipped with a fitted digital camera. All cells in three photos per slice for each bird were measured to obtain the adipocyte surface. Image analysis software Image-Pro Plus (Media Cybernetics, Inc., Bethesda, MD, USA) was used to measure the cross-sectional surface of the adipocytes.

#### Blood samples

Blood samples from six birds per treatment (one bird from each replicate) were collected in 21 d. The blood samples were collected from the wing vein of birds after a 12 h feed withdrawal. Serum was separated by centrifugation at 3,000×g for 15 min at 4°C. All samples were dispensed and kept at −20°C until further analysis. Serum samples were analyzed for the concentration of glucose, total cholesterol, triglyceride.

### Statistical analysis

The data was analyzed using the PROC MIXED procedure of SAS software (SAS Institute Inc., Cary, NC, USA). The values are means of the six replicates. Results were reported as least squares means. Tukey’s test was used to test the means separation. Contrasts were used to test the linear, quadratic and cubic changes affected due to the increasing amount of dietary FCSM. Significant differences were declared at p<0.05, and tendency were reported at 0.05≤p<0.10. The Logistic, Gompertz, and Bertalanffy growth models were used to fit the data of abdominal fat weight and subcutaneous fat thickness.

## RESULTS

### Growth performance

There was no significant difference on ADFI, ADG, or FCR among the treatments in both d 1 to 7, d 8 to 14, and d 22 to 28 as well as for the entire period of the trial (d 1 to 42). From 15 to 21 d, dietary FCSM linearly reduced the FCR (p<0.05) without linear nor quadratic trends on ADFI and ADG (p> 0.05). During the d 29 to 35, broiler chickens fed 3FCSM diet had higher ADFI (p<0.05) compared to those fed 9FCSM diet. Broiler chickens fed 9FCSM diet had lower ADG (p<0.05) compared to control and broiler chickens with 3FCSM and 6FCSM diets. There was no significant difference on FCR between treatments (p>0.05). In addition, ADFI and FCR decreased with a linear (p<0.05) fashion, but reacted with increasing dietary FCSM levels (Table 4) during the period from 36 to 42 days. Additionally, the diet cost of 0FCSM, 3FCSM, 6FCSM, and 9FCSM were 2.82, 2.89, 2.96, and 3.03, respectively and the cost of diet per kg of broiler meat produced were 5.14, 5.12, 5.34, and 5.44, respectively. The cost of FCSM per kg of broiler meat in 3FCSM, 6FCSM, and 9FCSM were 0.29, 0.59, and 0.89 RMB, respectively.

### Carcass trait

No significant differences on the percentage of eviscerated, semi-eviscerate, breast muscle, or thigh muscle were observed among all the dietary treatments at d 7 or 14 (p>0.05). The carcass yields linearly increased in response to dietary FCSM supplementation at d 14 (p<0.05). The percentage of carcass in groups fed on 9FCSM diet was lower than other groups (p<0.05) at d 21. At 28 d of age, dietary FCSM supplementation quadratically increased the percentage of semi-eviscerate (p<0.05). However, broiler chickens fed 3FCSM diet had higher percentage of eviscerate (p<0.05) compared with broiler chickens fed 9FCSM diet. In addition, broiler chickens fed 3FCSM diet had higher percentage of eviscerated (p<0.05) and semi-eviscerate (p < 0.05) compared with broiler chickens fed 6FCSM and 9FCSM diets at d 35. Breast muscle percentage was linearly decreased (p<0.05) with increasing FCSM levels. By the end of the experiment (d 42), the percentage of carcass increased in a quadratic (p<0.05) way among increasing FCSM in diets. The breast muscle content was significantly increased after feeding 3FCSM diet (p<0.05, Table 5).

### Fat deposition

**Figure 1:**
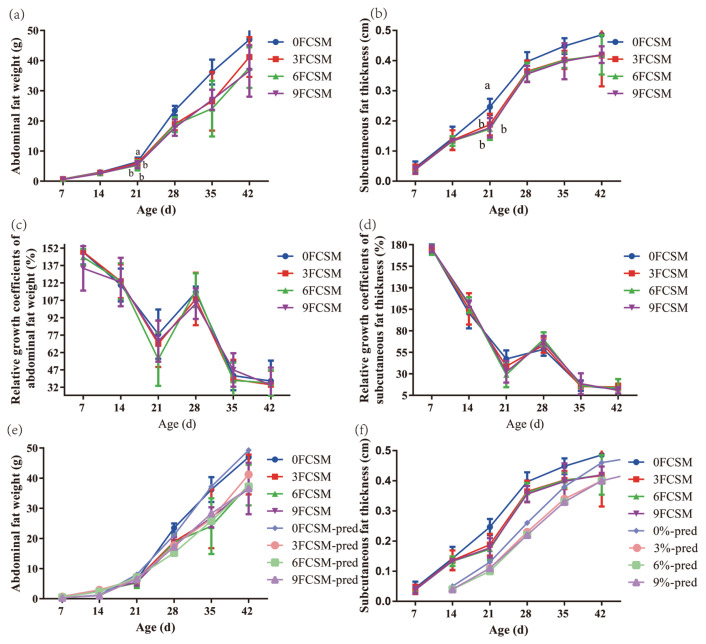
Effect of fermented cottonseed meal on fat deposition of broiler chickens. 0FCSM, 0% fermented cottonseed meal in diet; 3FCSM, 3% fermented cottonseed meal in diet; 6FCSM, 6% fermented cottonseed meal in diet; 9FCSM, 9% fermented cottonseed meal in diet. (a) Accumulative deposition curve of abdominal fat weight of broiler chickens. (b) Accumulative deposition curve of subcutaneous fat thickness of broiler chickens. (c) Relative growth coefficients of abdominal fat weight of broiler chickens. (d) Relative growth coefficients of subcutaneous fat thickness of broiler chickens. (e) Comparison between fitting growth curve of the Gompertz model and measured growth curve of abdominal fat weight. (f) Comparison between fitting growth curve of the Logistic model and measured growth curve of subcutaneous fat thickness.

Data show no effect of FCSM on the percentage of abdominal fat and subcutaneous fat thickness at d 7, 14, 28, 35, and 42. At d 21, the percentage of abdominal fat and subcutaneous fat thickness linearly decreased with increasing FCSM levels (p<0.05, Table 6). The growth of abdominal fat (Figure 1a) and subcutaneous fat (Figure 1b) presents an S-shaped curve, which is slower in the early stage and faster in the later stage. The relative growth coefficient of abdominal fat weight (Figure 1c) and subcutaneous fat thickness (Figure 1d) was the lowest at 14 to 21 d, during which the growth intensity of abdominal fat and subcutaneous fat thickness were the lowest.

Three models were used to fit the deposition law of abdominal fat weight and subcutaneous fat thickness with the change of age of the experimental white-feather broiler chickens. The R^2^ of Gompertz model was highest than the other two models for fitting the abdominal fat weight and in this case the fitting degree was the best (Table 7). Except that the actual value of abdominal fat weight of broiler chickens from 21 to 42 days old was slightly higher than that of the simulated curve, the measured growth curve of abdominal fat weight was in good agreement with the model fitting curve (Figure 1e). The Model Logistic has the highest R^2^ among the three models, which indicating that Model Logistic has the highest fitting degree for the subcutaneous fat growth curve of broiler chickens, even though the actual growth curve was slightly lower than the predicted curve at the age of 21 days (Figure 1f).

### Histopathological analysis and blood samples

According to the growth curve of abdominal fat and subcutaneous fat thickness, the relative growth coefficient of abdominal fat weight and subcutaneous fat thickness was the lowest at 14 to 21 d. Therefore, adipocytes and concentration of serum glucose, total cholesterol, triglyceride on 21 d were analysized.

**Figure 2:**
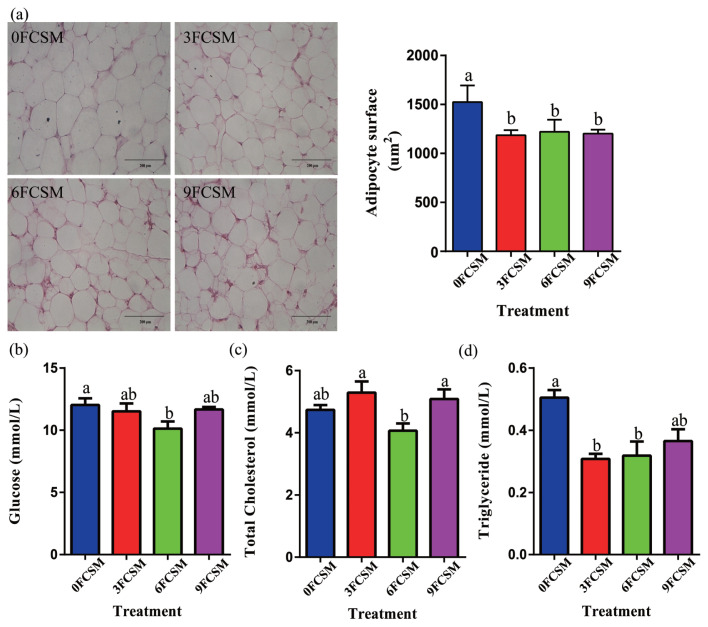
Effect of fermented cottonseed meal on adipocyte surface and serum lipid-related indices. 0FCSM, 0% fermented cottonseed meal in diet; 3FCSM, 3% fermented cottonseed meal in diet; 6FCSM, 6% fermented cottonseed meal in diet; 9FCSM, 9% fermented cottonseed meal in diet. (a) Adipocyte surface was demonstrated using hematoxylin-eosin (H&E) staining (Magnification, 100×; scale bar, 200 μm). (b) Serum glucose concentration. (c) Serum total cholesterol concentration. (d) Serum triglyceride concentration.

Compared with 0FCSM group, birds fed the FCSM-contained diet had smaller adipocytes in abdominal fat (p<0.05, Figure 2a). Glucose concentration was in 6FCSM group (p< 0.05, Figure 2b) compared with 0FCSM group (p<0.05, Figure 2b). No significant differences were observed on total cholesterol in 0FCSM group compared with others (p>0.05, Figure 2c). In addition, triglyceride concentration was decreased in 3FCSM and 6FCSM groups compared with 0FCSM group (p<0.05, Figure 2d).

## DISCUSSION

### Growth performance of broiler chickens

From the results of the present study, FCSM showed no effect on broiler chickens’ growth performance during d 1 to 7, d 8 to 14, d 22 to 28, and d 1 to 42. In this study, during the d 15 to 21, broiler chickens fed 6FCSM diet had lower FCR compared to those fed control diet. According to the result of present study, there were no significant differences in growth performance when broiler chickens ingested FCSM from 22 to 28 days of age. However, during the day of 29 to 35, broiler chickens fed 9FCSM diet had lower ADFI compared with broiler chickens fed 3FCSM diet and had the lowest ADG compared to other broiler chickens. Broiler chickens fed 9FCSM diets had lowest ADFI and FCR compared to other broiler chickens during the last week (36 to 42 days). Similarly, another research result showed that broiler chickens fed a diet with 12% FCSM had a lower ADG during the day of finisher (22 to 42 days) and the whole periods (1 to 42 days) than other treatments [1]. A study found that a small addition (7%) of fermented protein feedstuffs to pig diets resulted a positive effect on pig production and nutrient digestibility but with 7% fermented protein feedstuffs a negative effect was obtained [18].

The growth-promoting effects as mentioned above were considered to be mostly due to the improvement of nutritional value of the fermented feed. For instance, the microbial fermentation process can produce many beneficial substances such as small-size peptides, vitamins, organic acids, etc. [19]. Fermentation could improve the CP content as well as *in vitro* digestibility [12]. Another factor that cannot be ignored is the metabolic activity of microorganisms in the fermentation process which produces a number of enzymes namely, protease, amylase, lipase and phytase [20], which can be beneficial to the digestion of feed. As it is evident that a balanced microbial population would support a healthy intestinal tract functions [21]. Furthermore, FCSM could improve the intestinal digestive enzyme activity, intestinal morphology and bacterial ecology in broiler chickens [22], which may contribute to performance promotion. Additionally, due to the increased in the cost of FCSM, the cost of diets increased and the cost of diet per kg of broiler meat produced.

### Carcass traits

The supplement of FCSM in feed for broiler chickens has been reported before [1,3,4,7]. However, little research has been conducted to evaluate the effects of FCSM on slaughter performance of broiler chickens at different growth periods except for 21 and 42 days. The results of the current study showed that the percentage of carcass were higher than other treatments on day 14 and 42, when the broiler chickens fed 6FCSM diet. Interestingly, in the current research, the percentage of semi-eviscerate was improved by adding 3FCSM in the 21, 28 and 35 d growth trials and breast muscle was increased significantly on the day of 42. Improved carcass traits may be related with an increased performance in the growth of broiler chickens after adding FCSM. Jazi et al [23] showed that even up to 20%, FCSM could increase the carcass traits of broiler chickens. Notably, the increased percentage of breast muscle occurred when the broiler chickens were fed with 3FCSM diet. This might indicate the alter of anabolism of intramuscular protein.

### Fat deposition

With the increasing FCSM levels, abdominal fat and subcutaneous fat thickness linearly decreased on d 21. The results of fat deposition in the current study is consistent with the previous results from the same laboratory [4]. Studies showed that fermented feed produces reduced fat content. More specifically, Cha et al [10] showed that fermented soybean-based red pepper paste decreases visceral fat and improves blood lipid profiles in overweight adults. The results of Park et al [11] showed that kimchi fermented with the starter *Weissella koreensis* OK1-6 has anti-obesity effects in high-fat diet-induced obese mice. Furthermore, it has been seen that yeast probiotic-containing diets decrease the fat deposition of broiler chickens [24]. Therefore, the probiotics of FCSM may influence lipid metabolism [7]. Moreover, in our previous study, we found that the fermentation process produces varieties of metabolites included in the fermented substrate, such as L-carnitine, small peptide, nicotinic acid, phosphorylcholine, amino acid, and organic acid. According to literature, L-carnitine has strong effect of hypolipidemic and ameliorates dyslipidemic [25]. On the other hand, small peptides block fat absorption and promote lipid metabolism. It is, therefore, suggested that the effects of FCSM on the abdominal fat and subcutaneous fat thickness are also related with the metabolites during the fermentation process. In addition, birds fed the FCSM-contaminated diet had smaller adipocyte surface of abdominal fat in this study. It appears, hence, that FCSM prevented adipocyte hypertrophy, thereby reducing fat accumulation. In the present study, birds that received the 6FCSM diet on 21 d had lower glucose and triglyceride concentration. Glucose is one of the metabolites indicative of animal energy status. Triglyceride exists in lipid droplets, which is the main component of serum lipid. Therefore, it is suggested that FCSM might alleviate fat accumulation by affecting energy metabolism and triglyceride concentration of chickens.

Despite our increased understanding on how FCSM effects the animal growth performance, our knowledge of FCSM modulation of host fat deposition is limited. In particular, it is not clear how the FCSM effects the curve of fat deposition tissues such as abdominal and subcutaneous. In the current study, the accumulative growth curves of abdominal fat weight and subcutaneous fat were different, which was consistent with the study of Yang et al [26]. The difference of deposition between abdominal fat and subcutaneous fat, which were probably due to differences in hormone and enzyme activity in adipose tissue, as well as the development status of microvascular tissue. Supplementing FCSM in the diets of broiler chicken were failed to influence the shape of accumulative growth curves of abdominal fat weight and subcutaneous fat in broiler chickens. But the reduced height of the curves, indicating that the FCSM decreased the deposition of fat. The general trend of relative growth coefficient of abdominal fat weight and subcutaneous fat thickness decreased with increasing age, but there was a rapid deposition of abdominal fat and subcutaneous fat during the 21 to 35 period. It might be due to the number increasing in adipocytes of broilers before four weeks. This phenomenon over time leads to an increase in the fat cell size which finally became the main process of adipose tissue growth.

The growth curve equation is a mathematical model used to describe the relationship between animal growth and the age. It describes the growth pattern, body weight and parts and summarize the information into a few biologically interpretable parameters [27]. Information related to growth curves is important for the efficient production of animals. Linear and nonlinear growth models have been used to investigate growth of organism through a time interval. These are Gompertz, Logistic, Bertalanffy, Richard, Morgan-Mercer-Flodin, and Weibull models [28–30]. In our study, Logistic, Gompertz, and Bertalanffy were chosen to analyze the effect of FCSM on the growth of abdominal fat and subcutaneous fat. All these three growth model equations have been applied most frequently for poultry. The results of our study show that the Gompertz and Logistic functions provide a better fit on abdominal fat and subcutaneous fat, respectively. Moreover, the equations predict that the inflection point of abdominal fat and subcutaneous fat deposition was around 28 d, which was consistent with the accumulative growth curve of abdominal fat weight and subcutaneous fat thickness. In the present study, there were no significant differences in the inflection point of abdominal fat and subcutaneous fat deposition when broiler chicken intake FCSM.

In summary, supplementing FCSM to broiler chickens diet improved carcass trail and has different effects on growth performance of broilers at different ages. FCSM beneficially impacted the fat deposition by affecting adipocyte surface, serum glucose and triglyceride concentration of chickens. Furthermore, the Gompertz and Logistic functions provided a better fit on abdominal fat and subcutaneous fat, respectively. The equations predict that the inflection point of abdominal fat and subcutaneous fat deposition was around 28 d. So, the present study provides a practical approach for the application of FCSM as a functional feed and utilization of microbial fermented feedstuffs for poultry production. In addition, there are limitations in this study, such as the exact effects of fermentation metabolites and probiotics included in FCSM on lipid metabolism are unknown, and requires further research.

## Figures and Tables

**Table 1:** Ingredient compositions and nutrient contents of the experimental diets

Items (g/kg)	1 to 3 wk1)				4 to 6 wk1)			
	
	0FCSM	3FCSM	6FCSM	9FCSM	0FCSM	3FCSM	6FCSM	9FCSM
Ingredient								
Yellow corn	545.0	545.0	543.0	543.0	582.0	582.0	581.5	580.0
Soybean meal	335.0	304.0	274.0	243.0	288.0	257.0	226.5	196.0
Fermented cottonseed meal	0.0	30.0	60.0	90.0	0.0	30.0	60.0	90.0
Sunflower oil	30.0	31.0	33.0	34.0	40.0	41.0	42.0	44.0
Cottonseed protein	40.0	40.0	40.0	40.0	40.0	40.0	40.0	40.0
Premix2)	50.0	50.0	50.0	50.0	50.0	50.0	50.0	50.0
Nutrient content3)								
Metabolizable energy (Mcal/kg)	2.95	2.95	2.95	2.95	3.06	3.05	3.05	3.05
Crude protein	212.3	212.1	212.3	212.1	195.2	195.1	195.2	195.1
Ether extract	55.7	55.4	56.0	55.8	65.9	65.8	66.1	66.0
Crude ash	72.2	72.3	72.4	72.5	71.2	71.3	71.3	71.4
Crude fiber	31.1	31.7	32.3	32.9	29.1	29.7	30.3	30.9
Calcium	10.4	10.3	10.3	10.3	9.6	9.6	9.6	9.5
Total phosphorus	6.9	7.0	7.1	7.2	6.4	6.5	6.6	6.7
Available phosphate	4.5	4.5	4.5	4.6	4.1	4.1	4.1	4.2
Methionine	5.0	5.0	5.0	5.0	4.8	4.8	4.8	4.8
Methionine+cysteine	8.6	8.6	8.6	8.6	8.2	8.2	8.2	8.2
Threonine	7.8	7.7	7.6	7.4	7.1	7.0	6.9	6.7
Lysine	11.2	10.9	10.8	10.5	10.0	9.8	9.6	9.4
Free gossypol	16.0	17.1	18.2	19.3	16.0	17.1	18.2	19.3

1) 0FCSM, 0% fermented cottonseed meal in diet; 3FCSM, 3% fermented cottonseed meal in diet; 6FCSM, 6% fermented cottonseed meal in diet; 9FCSM, 9% fermented cottonseed meal in diet.

2) Premix provided the following per kg of diets: CaCO3 10.67 g (1 to 21 d), 11.34 g (21 to 42 d), CaHPO4 13.07 g (1 to 21 d), 9.8 g (21 to 42 d); NaCl 2.94 g, Lys 0.234 g, Met 1.791 g, retinol 8,800 IU, cholecalciferol 3,000 IU, ɑ-tocopherol 30 mg, menaquinone 1.65 mg, thiamin 2.5 mg, riboflavin 6.6 mg, nicotinic acid 11 mg, adenine 500 mg, pantothenic acid 60 mg, pyridoxine 4.0 mg, biotin 0.2 mg, folic acid 1.0 mg, cyanocobalamin 0.02 mg, ascorbic acid 50 mg, Fe 80.0 mg, Cu 8.0 mg, Zn 60.0 mg, Mn 70.0 mg, I 0.5 mg, Se 0.3 mg.

3) The nutrient levels are measured values except metabolizable energy and amino acid contents, which were calculated.

**Table 2:** Chemical composition of cottonseed meal, fermented cottonseed meal, soybean meal, and cottonseed protein

Item	CSM	FCSM	Soybean meal	Cottonseed protein
DM (%)	91.41	91.26	90.52	90.87
Crude protein (% of DM)	40.16	44.42	44.26	50.12
Ether extract (% of DM)	0.83	0.86	1.87	1.00
Crude ash (% of DM)	5.82	6.43	6.24	5.70
Acid detergent fiber (% of DM)	16.21	13.08	10.21	13.70
Neutral detergent fiber (% of DM)	26.13	21.78	13.52	20.00
Free gossypol (mg/kg)	150.10	36.41	-	400
Ca (% of DM)	0.28	0.29	0.30	0.29
P (% of DM)	0.69	0.72	0.68	0.22

CSM, cottonseed meal; FCSM, fermented cottonseed meal; DM, dry matter.

**Table 3:** The growth curve model and parameters1)

Model	Equation	Inflexion point weight (g/cm)	Maximum weekly gain (g/cm)	Inflexion age (wk)	Relative growth rate (%)	Absolute growth rate (%)
Logistic	Y = A/(1+Be^−kt^)	A/2	kW/2	(lnB)/k	k(1−W_i_/A)	W_i_k(1−W_i_/A)
Gompertz	Y = Ae^−Bexp(−kt)^	A/e	kW	(lnB)/k	k(lnA−lnW_i_)	W_i_k(lnA−lnW_i_))
Von Bertalanffy	Y = A(1−Be^−kt^)^3^	8A/27	3kW/2	(ln3B)/k	3k[(A/W_i_)^1/3^−1]	W_i_3k[(A/W_i_)^1/3^−1]

1) Y is the expected abdominal fat weight or subcutaneous fat thickness at the week of t; A is the maximum abdominal fat weight or subcutaneous fat thickness when age approaches infinity; k is the coefficient of relative growth or maturing index; B is the integration constant which is related to hatching weight or thickness; t is time (age); W is the inflection point weight; W_i_ is the weight in week i.

**Table 4:** Effect of fermented cottonseed meal on the growth performance of broiler chickens

Items	Treatments1)				SEM	p-value			
	
	0FCSM	3FCSM	6FCSM	9FCSM		Treatment	Linear	Quadratic	Cubic
d 1 to 7									
ADFI (g/d)	17.98	20.28	16.66	17.03	1.79	0.81	0.66	0.69	0.44
ADG (g/d)	11.98	13.03	11.35	11.01	1.44	0.39	0.06	0.06	0.49
FCR (g/g)	1.62	1.56	1.48	1.59	0.31	0.32	0.06	0.06	0.17
Mortality rate (%)2)	1.70	0.89	1.70	1.70	1.39	0.98	-	-	-
d 8 to 14									
ADFI (g/d)	41.36	41.48	41.61	42.16	1.30	0.85	0.97	0.65	0.46
ADG (g/d)	23.79	24.58	24.69	24.87	0.79	0.22	0.64	0.06	0.47
FCR (g/g)	1.74	1.68	1.68	1.69	0.07	0.34	0.12	0.07	0.87
Mortality rate (%)	0.89	0.00	0.89	0.00	1.01	0.76	-	-	-
d 15 to 21									
ADFI (g/d)	64.61	60.02	54.32	53.11	6.58	0.74	0.33	0.65	0.91
ADG (g/d)	38.02	41.05	42.09	37.82	2.24	0.87	0.49	0.76	0.76
FCR (g/g)	1.69^a^	1.46^ab^	1.29^b^	1.41^ab^	0.16	0.04	0.04	0.06	0.99
Mortality rate (%)	0.00	0.93	0.00	0.00	-	-	-	-	-
d 22 to 28									
ADFI (g/d)	97.73	103.6	93.85	91.67	6.69	0.76	0.46	0.84	0.44
ADG (g/d)	45.40	55.33	50.59	47.36	3.52	0.48	0.82	0.64	0.20
FCR (g/g)	2.15	1.87	1.85	1.94	0.21	0.49	0.50	0.39	0.42
Mortality rate (%)	1.00	1.00	0.00	1.00	0.37	0.88	-	-	-
d 29 to 35									
ADFI (g/d)	111.15^ab^	124.24^a^	120.88^ab^	99.13^b^	7.78	0.05	0.19	0.03	0.91
ADG (g/d)	74.54^a^	76.43^a^	74.50^a^	57.72^b^	4.38	0.03	0.05	0.10	0.55
FCR (g/g)	1.71	1.63	1.62	1.72	0.12	0.55	0.35	0.35	0.64
Mortality rate (%)	0.0	0.0	0.0	0.0	-	-	-	-	-
d 36 to 42									
ADFI (g/d)	140.78^a^	141.97^a^	137.13^a^	106.99^b^	5.75	0.01	0.01	0.06	0.38
ADG (g/d)	70.19	77.06	75.66	60.62	5.26	0.81	0.87	0.23	0.29
FCR (g/g)	2.01^a^	1.85^ab^	1.81^ab^	1.75^b^	0.12	0.08	0.03	0.07	0.07
Mortality rate (%)	1.18	2.29	1.18	1.18	1.05	0.59	-	-	-
d 1 to 42									
ADFI (g/d)	78.93	81.93	76.14	70.78	6.81	0.59	0.22	0.55	0.83
ADG (g/d)	43.23	46.17	42.18	39.42	4.90	0.13	0.06	0.19	0.66
FCR (g/g)	1.83	1.78	1.80	1.80	0.04	0.56	0.75	0.16	0.94
Cumulative mortality rate (%)	4.17	4.17	3.30	3.30	2.32	0.62	-	-	-
Cost of diet (YUAN/kg)	2.82	2.82	2.82	2.82	-	-	-	-	-
Cost of diet per kg of broiler meat (YUAN/kg)	5.14	5.00	5.09	5.06	-	-	-	-	-
Cost of FCSM (YUAN/kg)	3.2	-	-	-	-	-			
The meat production affected by FCSM (kg)	0	0.05	0.11	0.16	-	-	-	-	-

1) 0FCSM, 0% fermented cottonseed meal in diet; 3FCSM, 3% fermented cottonseed meal in diet; 6FCSM, 6% fermented cottonseed meal in diet; 9FCSM, 9% fermented cottonseed meal in diet. The values are means of the six replicates.

SEM, standard error of the mean; ADFI, average daily feed intake; ADG, average daily weight gain; FCR, feed conversion ratio (ADFI/ADG).

2) Mortality rates, mortality during the subsequent periods was based on the number alive in the remaining pens at the start of each period.

a,b Means within the same row with different lowercase letters indicate significant difference (p<0.05).

**Table 5:** Effect of fermented cottonseed meal on the carcass traits of broiler chickens (%)

Item	Treatments1)				SEM	p-value			
	
	0FCSM	3FCSM	6FCSM	9FCSM		Treatment	Linear	Quadratic	Cubic
d 7									
Carcass	87.32	89.79	88.54	88.56	1.45	0.26	0.26	0.42	0.14
Eviscerated	57.85	57.57	58.10	57.38	0.88	0.24	0.79	0.41	0.07
Semi-eviscerate	82.06	82.79	84.17	82.91	1.48	0.49	0.38	0.31	0.45
Liver	7.16	7.34	6.99	7.19	0.19	0.06	0.43	0.18	0.16
Breast muscle	15.40	14.67	14.99	14.01	0.80	0.81	0.42	0.90	0.62
Thigh muscle	16.34	16.97	17.08	17.32	0.60	0.13	0.23	0.22	0.09
d 14									
Carcass	87.47^b^	87.07^b^	89.55^a^	87.87^b^	0.35	0.01	0.01	0.05	0.02
Eviscerated	60.52	60.59	59.69	57.33	1.65	0.26	0.08	0.35	0.93
Semi-eviscerate	79.93	79.68	79.02	77.82	0.39	0.24	0.05	0.54	0.97
Liver	5.88	5.57	5.21	5.57	0.42	0.27	0.22	0.16	0.48
Breast muscle	16.8	16.73	17.96	18.34	0.96	0.31	0.13	0.57	0.34
Thigh muscle	16.79	16.95	16.39	16.67	0.43	0.98	0.82	0.95	0.71
d 21									
Carcass	88.93^a^	88.95^a^	88.21^a^	86.44^b^	0.23	0.02	0.02	0.03	0.19
Eviscerated	69.96	72.86	69.60	70.9	1.58	0.19	0.93	0.48	0.05
Semi-eviscerate	85.44^b^	89.77^a^	86.74^ab^	87.76^ab^	0.95	0.02	0.47	0.13	0.05
Liver	3.81	4.18	4.12	4.21	0.30	0.49	0.23	0.43	0.53
Breast muscle	22.27	22.34	22.46	22.73	0.47	0.99	0.74	0.92	0.99
Thigh muscle	16.83	18.21	17.17	18.37	0.93	0.54	0.37	0.92	0.25
d 28									
Carcass	87.80	88.11	87.02	87.02	0.51	0.99	0.88	0.91	0.91
Eviscerated	68.94^ba^	73.10^a^	71.66^ab^	68.71^b^	0.58	0.01	0.62	0.01	0.34
Semi-eviscerate	81.85^b^	87.39^a^	87.59^a^	83.34^ab^	0.92	0.05	0.53	0.01	0.90
Liver	4.50	4.31	4.51	4.56	0.15	0.73	0.62	0.48	0.46
Breast muscle	25.83	25.94	24.07	24.59	1.21	0.67	0.34	0.87	0.45
Thigh muscle	17.15	18.28	18.30	17.82	1.13	0.78	0.62	0.38	0.88
d 35									
Carcass	87.07	88.33	88.24	87.06	1.36	0.29	0.07	0.76	0.59
Eviscerated	70.70^ab^	74.55^a^	69.82^b^	69.83^b^	1.75	0.05	0.24	0.17	0.04
Semi-eviscerate	85.54^b^	89.80^a^	84.95^b^	84.64^b^	1.25	0.04	0.68	0.53	0.01
Liver	4.21	3.93	4.27	4.27	0.17	0.35	0.45	0.36	0.17
Breast muscle	23.18^a^	22.94^a^	20.6^b^	19.42^b^	0.48	0.01	0.01	0.55	0.35
Thigh muscle	21.51	21.49	20.12	20.21	0.88	0.39	0.14	0.95	0.42
d 42									
Carcass	87.48^b^	90.82^a^	90.69^a^	88.76^ab^	1.34	0.05	0.18	0.01	0.55
Eviscerated	75.94	76.43	74.99	73.57	1.54	0.27	0.09	0.38	0.68
Semi-eviscerate	90.07	90.81	90.14	89.87	0.71	0.97	0.85	0.74	0.79
Liver	3.17	3.36	3.46	3.56	0.30	0.59	0.19	0.83	0.92
Breast muscle	23.50^b^	25.86^a^	22.29^b^	22.85^b^	0.45	0.04	0.16	0.18	0.02
Thigh muscle	21.99	22.63	21.83	22.79	0.46	0.77	0.64	0.83	0.36

SEM, standard error of the mean.

1) 0FCSM, 0% fermented cottonseed meal in diet; 3FCSM, 3% fermented cottonseed meal in diet; 6FCSM, 6% fermented cottonseed meal in diet; 9FCSM, 9% fermented cottonseed meal in diet. The values are means of the six replicates.

a,b Means within the same row with different lowercase letters indicate significant difference (p<0.05).

**Table 6:** Effect of fermented cottonseed meal on the abdominal fat (% of eviscerated weight) and subcutaneous fat thickness (cm)

Items	Treatments1)				SEM	p-value			
	
	0FCSM	3FCSM	6FCSM	9FCSM		Treatment	Linear	Quadratic	Cubic
d 7									
Abdominal fat	1.08	0.98	0.97	0.96	0.07	0.84	0.45	0.69	0.87
Subcutaneous fat thickness	0.04	0.04	0.04	0.04	0.00	0.89	0.46	0.75	0.99
d 14									
Abdominal fat	1.75	1.62	1.45	1.46	0.17	0.34	0.09	0.63	0.70
Subcutaneous fat thickness	0.14	0.13	0.13	0.13	0.01	0.95	0.60	0.77	0.99
d 21									
Abdominal fat	1.64^a^	1.55^b^	1.53^b^	1.53^b^	0.03	0.04	0.02	0.83	0.97
Subcutaneous fat thickness	0.25^a^	0.19^b^	0.17^b^	0.18^b^	0.01	0.01	0.01	0.05	0.72
d 28									
Abdominal fat	3.78	3.12	3.17	3.21	0.43	0.43	0.21	0.24	0.55
Subcutaneous fat thickness	0.40	0.36	0.36	0.36	0.03	0.53	0.75	0.41	0.24
d 35									
Abdominal fat	3.76	3.31	3.18	3.36	0.29	0.71	0.49	0.46	0.99
Subcutaneous fat thickness	0.45	0.40	0.40	0.40	0.01	0.15	0.06	0.23	0.54
d 42									
Abdominal fat	3.66	3.14	3.13	3.17	0.18	0.72	0.39	0.49	0.81
Subcutaneous fat thickness	0.49	0.42	0.42	0.42	0.02	0.38	0.19	0.29	0.61

SEM, standard error of the mean.

1) 0FCSM, 0% fermented cottonseed meal in diet; 3FCSM, 3% fermented cottonseed meal in diet; 6FCSM, 6% fermented cottonseed meal in diet; 9FCSM, 9% fermented cottonseed meal in diet. The values are means of the six replicates.

a,b Means within the same row with different lowercase letters indicate significant difference (p<0.05).

**Table 7:** Fitting degree and parameters evaluation of grow curves of three curve models for abdominal fat weight and subcutaneous fat thickness of broiler chickens

Items	Model type	Treatment1)	Parameter2)			R^2^	Inflexion point weight (g) or thickness (cm)	Maximum weekly gain (g or cm)	Inflexion point age (wk)

			A (g/cm)	B	k (%)				
Abdominal fat (g)	Logistic	0FCSM	55.25	248.00	1.26	0.994	27.63	17.40	4.38
		3FCSM	59.18	107.73	0.91	0.993	29.59	13.46	5.14
		6FCSM	50.88	106.53	0.94	0.984	25.44	11.96	4.97
		9FCSM	40.06	248.45	1.30	0.997	20.03	13.02	4.24
	Gompertz	0FCSM	55.00	24.36	0.81	0.996	20.22	16.38	3.94
		3FCSM	50.00	24.15	0.80	0.996	18.38	14.71	3.98
		6FCSM	48.00	24.51	0.82	0.987	17.65	14.47	3.90
		9FCSM	45.38	24.28	0.81	0.996	16.68	13.51	3.94
	Bertalanffy	0FCSM	89.85	1.64	0.37	0.994	26.62	14.78	4.31
		3FCSM	80.00	1.01	0.27	0.996	23.70	9.60	4.11
		6FCSM	71.42	1.07	0.26	0.988	21.16	8.25	4.49
		9FCSM	62.19	1.66	0.39	0.994	18.43	10.78	4.12
Subcutaneous fat thickness (cm)	Logistic	0FCSM	0.50	35.98	0.93	0.996	0.25	0.12	3.85
		3FCSM	0.44	38.89	1.00	0.977	0.22	0.11	3.66
		6FCSM	0.45	36.54	0.91	0.971	0.23	0.10	3.95
		9FCSM	0.44	38.92	0.99	0.975	0.22	0.11	3.70
	Gompertz	0FCSM	0.54	6.08	0.46	0.994	0.20	0.09	3.92
		3FCSM	0.48	6.05	0.45	0.969	0.18	0.08	4.00
		6FCSM	0.49	6.11	0.46	0.962	0.18	0.08	3.93
		9FCSM	0.49	5.94	0.47	0.968	0.18	0.08	3.79
	Bertalanffy	0FCSM	0.58	1.39	0.37	0.992	0.17	0.10	3.86
		3FCSM	0.51	1.37	0.36	0.965	0.15	0.08	3.93
		6FCSM	0.53	1.29	0.36	0.958	0.16	0.08	3.76
		9FCSM	0.53	1.27	0.35	0.964	0.16	0.08	3.82

1) 0FCSM, 0% fermented cottonseed meal in diet; 3FCSM, 3% fermented cottonseed meal in diet; 6FCSM, 6% fermented cottonseed meal in diet; 9FCSM, 9% fermented cottonseed meal in diet. The values are means of the six replicates.

2) A is the maximum abdominal fat weight or subcutaneous fat thickness when age approaches infinity; B is the integration constant which is related to hatching weight or thickness; k is the coefficient of relative growth or maturing index.
